# Blinatumomab-Related Lineage Switch of KMT2A/AFF1-Rearranged B-Lymphoblastic Leukemia to B/Myeloid Mixed-Phenotype Acute Leukemia and Myeloid Sarcoma Causing Spinal Cord Compression

**DOI:** 10.1155/crh/7063561

**Published:** 2025-11-24

**Authors:** Xiaoming Fan, Ankita Gupta, Dominik Dabrowski, Mirza Rusella, Qihui J. Zhai, Eric X. Wei

**Affiliations:** ^1^Department of Pathology and Translational Pathobiology, LSU Health Shreveport, Shreveport, Louisiana 71103, USA; ^2^Department of Hematology and Oncology, Ochsner Health, 2700 Napoleon Ave, New Orleans, Louisiana 70115, USA; ^3^Department of Psychiatry, Rutgers University, Newark, New Jersey 07103, USA; ^4^Department of Pathology & Cell Biology, Tampa General Hospital, Ruffolo, Hooper, and Associates, University of South Florida, Tampa, Florida 33606, USA

## Abstract

Blinatumomab is a promising monoclonal antibody therapeutic for the treatment of relapsed or refractory B-cell precursor acute lymphoblastic leukemia (B-ALL). However, it has been associated with lineage switch in acute leukemia, particularly in cases of KMT2A-rearranged B-ALL, which carries a poor prognosis. While most lineage switch events present as acute myeloid leukemia (AML), rare cases may manifest as myeloid sarcoma. In the current report, we describe the development of myeloid sarcoma following blinatumomab treatment in a patient with refractory KMT2A-rearranged B-ALL. The patient, a 19-year-old African American male with primary Philadelphia chromosome-negative B-ALL, was initially treated with a pediatric-inspired chemotherapy regimen. He achieved pathologically morphologic remission after induction, but his minimal residual disease (MRD) testing remained persistently positive, eventually progressing to bone marrow relapse. The patient was then started on blinatumomab therapy, achieving a second morphological remission; however, his MRD remained detectable. During the fourth cycle of blinatumomab, the patient developed back pain and lower extremity weakness. Imaging revealed an extradural mass in the thoracic spine, resulting in spinal cord compression. Histopathologic evaluation of the mass confirmed a diagnosis of mixed-phenotype myeloid sarcoma harboring the same KMT2A rearrangement. Concurrent bone marrow biopsy revealed mixed-phenotype acute leukemia. The patient was subsequently lost to follow-up. This is the fourth reported case of myeloid sarcoma following blinatumomab therapy of persistent B-ALL. It highlights an unusual and serious pattern of relapse in an extramedullary site following blinatumomab therapy. Clinicians should remain vigilant for signs of lineage switch and extramedullary disease during treatment, particularly in patients with KMT2A-rearranged B-ALL, and consider imaging or biopsy when new neurologic or systemic symptoms arise.

## 1. Introduction

Rearrangements of the histone lysine [K]-methyltransferase 2A gene (KMT2A), formerly known as the mixed-lineage leukemia (MLL) gene and located on chromosome 11q23, are commonly observed in acute lymphoblastic leukemia (ALL). These rearrangements are associated with an increased risk of lineage switch during therapy and serve as an independent adverse prognostic factor. However, the mechanisms underlying such lineage conversions remain poorly understood.

Blinatumomab, the first and only Food and Drug Administration (FDA)–approved bispecific T-cell engager monoclonal antibody, binds to CD3 on T cells and facilitates recognition of CD19 on malignant B cells, thereby promoting immune-mediated cytolysis. It was approved for the treatment of relapsed or refractory precursor B-cell precursor acute lymphoblastic leukemia (B-ALL) in 2014 and subsequently in 2018 for patients in first or second remission with minimal residual disease (MRD) [1]. Nonetheless, an increasing number of reports have described cases of lineage switch associated with blinatumomab therapy. Most of these cases involve transformation to acute myeloid leukemia (AML) or myelodysplastic syndrome (MDS) [2–4], with only a limited number of cases presenting as myeloid sarcoma.

Here, we present a rare case of lineage switch manifesting as thoracic spinal myeloid sarcoma with spinal cord compression following blinatumomab therapy in a 19-year-old patient with refractory B-ALL with KMT2A rearrangement. This case highlights the critical need for further investigation into the mechanisms of blinatumomab resistance and underscores the importance of developing more effective therapies targeting MLL rearrangements, which remain associated with poor clinical outcomes.

## 2. Case Presentation

**Figure 1:**
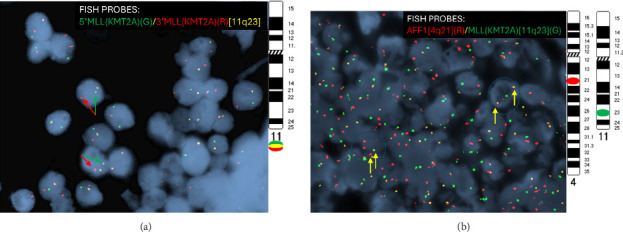
Fluorescence in situ hybridization (FISH) analysis of bone marrow samples. (a) MLL (KMT2A) break-apart FISH assay shows tumor cells positive for mixed-lineage leukemia rearrangement (MLL-r), with arrows indicating signal separation. (b) AFF1/MLL (KMT2A) dual color, dual fusion FISH assay demonstrates tumor cells positive for the KMT2A-AFF1 translocation, with arrows indicating fusion signals.

A 19-year-old African American male with no significant past medical history presented with epistaxis. Initial laboratory evaluation revealed severe thrombocytopenia (platelet count < 10,000/μL) and marked leukocytosis (WBC > 45,000/μL). Peripheral blood flow cytometry and bone marrow biopsy (100% cellularity) demonstrated a blast population positive for CD19, TdT, CD38, CD79a, and HLA-DR and negative for MPO, CD10, CD13, CD20, CD22, CD33, and CD34—consistent with a diagnosis of precursor B-ALL. Cytogenetic analysis identified an abnormal male karyotype with t (4; 11) (q21; q23): 46, XY, t (4; 11) (q21; q23)[5]/46, XY [15]. Fluorescence in situ hybridization (FISH) for the ALL panel was positive for MLL rearrangement (MLL-r, Figure 1(a)), and dual-color, dual-fusion FISH confirmed a KMT2A-AFF1 translocation (Figure 1(b)). The patient was started on a pediatric-inspired induction regimen comprising vincristine, doxorubicin, methotrexate, L-asparaginase, corticosteroids, and intrathecal cytarabine and methotrexate. This was followed by prophylactic cranial irradiation and cytarabine-based consolidation therapy.

**Figure 2:**
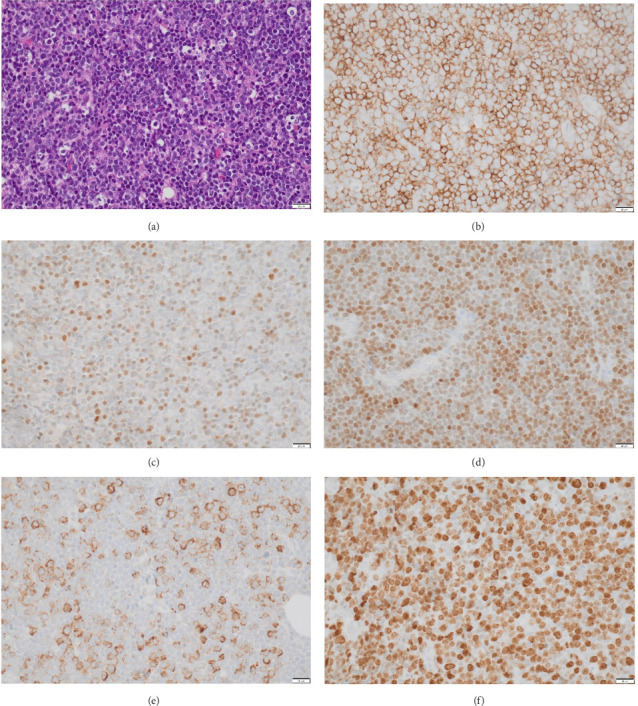
Histopathological examination of the epidural mass. The neoplasm demonstrates a hypercellular lesion with a characteristic “starry-sky” appearance (a), and immunochemistry showed that the tumor cells are positive for CD19 (b), TdT (c), PAX 5 (d), myeloperoxidase (e) with a high mitotic index (Ki-67, (f)). The scale bar indicates 20 μm.

**Figure 3:**
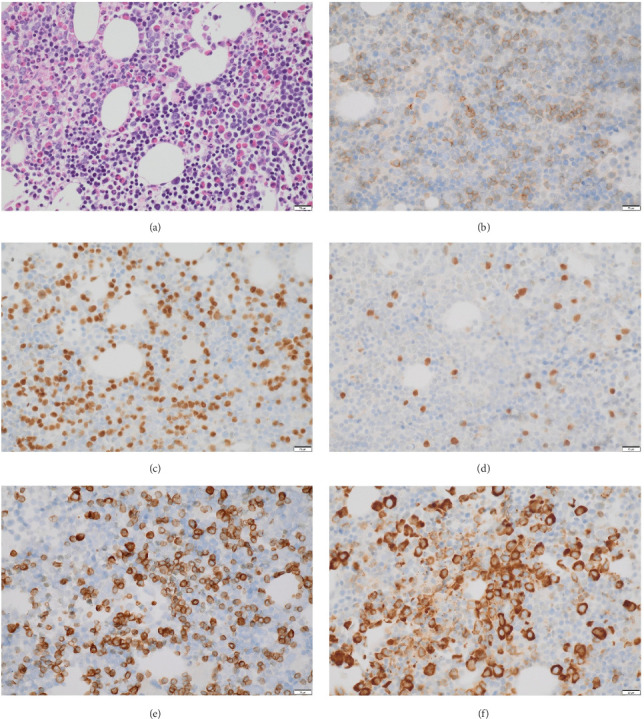
Concurrent bone marrow biopsy (a) demonstrated 15%–35% involvement by CD19 (b), PAX5 (c), TdT (d), and CD79a (e) positive blasts. MPO positivity (f) indicated an increased myelocytic component. The scale bar indicates 20 μm.

Serial bone marrow biopsies performed at 1, 3, 6, and 7 months postinduction showed morphologic remission. However, MRD analysis consistently detected CD19+ phenotypically abnormal cells, though MLL-r was not detected during this period. A subsequent bone marrow biopsy revealed 41% CD19+ blasts with recurrent MLL-r positivity, indicating disease progression. Salvage therapy with blinatumomab was initiated, leading to morphologic remission after the first cycle. Following the fourth cycle of blinatumomab, the patient presented with back pain and lower extremity weakness. MRI demonstrated an extradural mass extending from T3 to T5–6, causing spinal cord compression, along with a right paravertebral mass extending from T3–4 to T9. The patient underwent a laminectomy for extradural mass biopsy and partial resection of the mass to relieve symptoms. Histopathological examination revealed a malignant hematopoietic neoplasm with diffuse infiltration and numerous tangible body macrophages, creating a characteristic “starry-sky” appearance. Tumor cells were small to intermediate in size, with open chromatin, irregular nuclear contours, and a high nuclear-to-cytoplasmic (N:C) ratio. Immunohistochemistry showed positivity for PAX5, TdT, CD19, CD79a, and partial MPO expression (∼15% of cells) and negativity for CD1a, CD20, and CD34. The Ki-67 proliferation index was 100%, indicating a highly proliferative tumor (Figure 2). Flow cytometry findings were consistent with the immunophenotypic profile. Molecular analysis of the tumor cells revealed the same KMT2A rearrangement as previously identified. A diagnosis of myeloid sarcoma with mixed-phenotype features was rendered. A concurrent bone marrow biopsy demonstrated 15%–35% involvement by TdT+, CD19+, PAX5+, and CD79a + blasts. MPO positivity indicated an increased myelocytic component, indicating transformed mixed-phenotype acute leukemia (MPAL) (Figure 3). Molecular studies confirmed the presence of the t (4; 11) (q21; q23) translocation, consistent with previous findings. He subsequently underwent salvage therapy with the FLAG-Ida regimen (fludarabine, cytarabine, G-CSF, and idarubicin), followed by radiation to the thoracic spine. A postradiation bone marrow biopsy demonstrated residual disease. He was further evaluated for CD19-directed CAR T-cell therapy but was subsequently lost to follow-up.

## 3. Discussion

In this article, we report a rare case of lineage switch and development of myeloid sarcoma following both conventional chemotherapy and blinatumomab treatment in a 19-year-old patient with B-ALL. This case underscores the importance of continuous monitoring during treatment and highlights the urgent need to elucidate the mechanisms driving lineage switching in such patients.

B-ALL is a neoplasm of precursor lymphoid cells committed to the B-cell lineage, characterized by the expression of CD19, CD22, cytoplasmic CD79a, and/or PAX5, as determined by flow cytometry or immunohistochemistry [5]. It typically involves the bone marrow and frequently presents with peripheral blood involvement. B-ALL accounts for approximately 10% of lymphoblastic lymphoma cases and demonstrates a bimodal age distribution, with peaks in childhood and again around the fifth decade of life [6–9]. The current World Health Organization (WHO) classification of ALL relies heavily on cytogenetic and molecular abnormalities, which are critical determinants of prognosis [5].

KMT2A-rearranged ALL is a rare but aggressive subtype, comprising approximately 5% of ALL cases [10–12]. Over 90 fusion partners of KMT2A have been identified [13], with AFF1 on chromosome 4q21 being the most frequent, accounting for up to 84% of KMT2A rearrangements in adults and 44%–49% in infants and children [10, 13].

Lineage switch is a known complication in B-ALL management, particularly in KMT2A-rearranged cases [14]. Although rare, it occurs in approximately 6% of all relapsed childhood leukemias [15] and in about 7% of KMT2A-rearranged B-ALL cases [4]. Lineage switching is associated with poor survival outcomes, and current therapeutic strategies have shown limited success [4, 16]. A deeper understanding of the underlying mechanisms is urgently needed to inform the development of preventative and therapeutic strategies.

Most lineage switches in B-ALL result in AML or MDS [2–4]. Only a few cases of lineage switch presenting as myeloid sarcoma have been reported (Table 1) [4, 17–19], with varied sites of involvement including the tonsils, breast, or multiple locations. Immunophenotypically, three cases showed a complete lineage switch to myeloid sarcoma, characterized by the complete loss of CD19 and gain of MPO. In contrast, one case initially presented with a mixed-phenotype myeloid sarcoma (CD19 retention with partial MPO acquisition) at first relapse, which then evolved into a complete myeloid sarcoma at second relapse. Our case aligns with the latter presentation, manifesting as a mixed-phenotype myeloid sarcoma in a thoracic extradural mass causing spinal cord compression.

The mechanisms behind lineage switching remain poorly understood. Most reported cases involve KMT2A rearrangements, particularly in pediatric and infant populations [3, 4, 14]. Consistent with these observations, our patient was diagnosed with B-ALL at age 19 and experienced lineage switch at age 20. KMT2A rearrangement is associated with MPAL, featuring both lymphoid and myeloid characteristics [20]. Recent advances in molecular analysis, particularly single-cell sequencing, have begun to shed light on the mechanisms of lineage switching in this context [21, 22]. Epigenetic alteration and microenvironment are at the center of those hypothesized mechanisms. In the study by Tirtakusuma et al. [23], changes in chromatin accessibility and transcription factor binding site occupancy were observed during lineage switching in B-ALL with KMT2A rearrangements. These alterations may lead to transcriptional reprogramming, shifts in gene expression profiles, and modifications in immunophenotyping, thereby disrupting existing lymphoid or myeloid differentiation pathways and ultimately driving lineage switching. Specifically, fusion proteins resulting from KMT2A rearrangements cause constitutive and aberrant activation of the homeobox A (HOXA) gene cluster, thereby locking cells in an undifferentiated state. This transcriptional program is sustained by disruptor of telomeric silencing 1-like (DOT1L), a histone methyltransferase that is recruited by the fusion proteins to deposit crucial histone H3 lysine 79 (H3K79) methylation marks on target promoters. This epigenetic modification perpetuates the aberrant gene expression, which supports leukemic cell survival and culminates in a lineage switch [24].

Notably, many reported cases of lineage switch in B-ALL occurred either during or shortly after induction therapy, particularly in patients receiving CD19-directed immunotherapies such as blinatumomab or CD19 CAR T-cell therapy [4, 14]. Blinatumomab has demonstrated promising efficacy in refractory ALL, with remission rates of up to 50% [25]. However, lineage switch associated with blinatumomab use has increasingly been reported [4, 26–28], sometimes occurring as early as 9 days after treatment initiation [28].

Possible explanations for lineage switching following blinatumomab may be partly attributable to the plasticity of KMT2A-rearranged leukemia. In the study by Chen et al. [21], the authors proposed that KMT2A-rearranged leukemia originates from an uncommitted precursor with high lineage infidelity and the capacity for both lymphoid and myeloid differentiation. During CD19-directed immunotherapies such as blinatumomab, the lymphoid subpopulations may respond and derive therapeutic benefits. However, myeloid-primed subpopulations present at diagnosis may act as a reservoir, enabling lineage switching under the selective pressure of B-cell–directed immunotherapy.

Our patient, who had a KMT2A rearrangement at initial diagnosis, achieved KMT2A rearrangement negativity in the bone marrow after the first cycle of blinatumomab. However, MRD positivity with CD19+ blasts persisted, and the disease later relapsed as CD19+ B-ALL with concurrent myeloid sarcoma harboring KMT2A rearrangement in the thoracic spinal cord. This suggests a myeloid transformation of a blinatumomab-resistant CD19+ clone, leading to extramedullary relapse under immunotherapeutic pressure.

B-ALL with clonal evolution represents another possible explanation for the observations in the present case. Compared with B-ALL with lineage switch, clonal evolution has been reported only rarely in the literature. The molecular abnormalities described in such cases include additional chromosomal aberrations on the background of t (4; 11) (q21; q23) (KMT2A rearrangements) after chemotherapy [29] or a del (9) (p13p21) abnormality [19]. Lineage switch, although uncommon, has been reported more frequently and is often associated with KMT2A rearrangements. In the current case, while MPAL arising from clonal evolution could also be considered, we retained the terminology of lineage switch in this report. In our opinion, both clonal evolution and lineage switch are associated with poor prognosis, greater disease complexity following treatment, and underlying baseline molecular abnormalities. However, further studies are warranted to explore this topic in greater depth.

Collectively, these findings raise concerns regarding the use of blinatumomab as a monotherapy in KMT2A-rearranged ALL. The treatment for lineage switch following immunotherapy is not yet standardized. According to Project EVOLVE (Evaluation of Lineage Switch: An International Initiative) [30], cases presenting as AML are most commonly managed with an AML-induction chemotherapy regimen or the incorporation of gemtuzumab. However, given the rarity of lineage switch manifesting as myeloid sarcoma, an optimal treatment strategy remains undefined. Large-scale surveys and further investigation are needed to establish the most effective therapeutic approaches for this rare presentation.

## Figures and Tables

**Table 1:** Characteristics of the current case and previously reported lineage switch presenting with extramedullary relapse.

Case #	Gender	Age	Initial diagnosis	Treatment prior to relapse	Extramedullary relapse	Positive marker(s)	Concurrent bone marrow biopsy	Outcome	Reference
1	Male	34	B-ALL t (4:11)/KMT2A::AFF1	HCVAD × 4, mHCVD, inotuzumab × 3, Allo-SCT	MS × 2 (left tonsillar region)	Not specified	Lymphoid blasts in bone marrow	Full marrow involvement, no further treatment	[4]
2	Female	27	B-ALL t (4:11)/KMT2A::AFF1	CALGB 10403, mHCVD × 3, inotuzumab, blinatumomab, Allo-SCT	MS (multiple, location not specified)	Not specified	No involvement	Sarcoma shrunk after treatment	[4]
3	Female	40	B-ALL t (4:11)/KMT2A::AFF1	HCVAD, blinatumomab	MS × 2 (breast)	Lysozyme	AML with monocytic differentiation	Passed away	[17]
4	Male	14	B-ALL 46, XY, del (9) (p13p21)	Four-drug induction chemotherapy	MS with mixed phenotype (Testis)	MPO, PAX5, CD19	No involvement	Multiple relapses and passed away	[19]
2nd relapse					MS (left gluteal)	MPO, CD33, CD43, CD56, CD68	MPAL (myeloid major)		
5 (Current)	Male	19	B-ALL t (4:11)/KMT2A::AFF1	Pediatric-inspired regimens, blinatumomab × 4	MS with mixed phenotype (Epidural)	MPO, PAX5, CD19	MPAL	Lost to follow-up	Current

Abbreviations: Allo-SCT, allogeneic stem cell transplantation; AML, acute myeloid leukemia; CD, cluster of differentiation; HCVAD, hyperfractionated cyclophosphamide, vincristine, doxorubicin, and dexamethasone; mHCVD, mini-hyperfractionated cyclophosphamide, vincristine, and dexamethasone; MPAL, mixed-phenotype acute leukemia; MPO, myeloperoxidase; MS, myeloid sarcoma; PAX5, paired box 5.

## Data Availability

The clinical and histopathological data used to support the findings of this study are included within the article.

## Supporting Information

Supporting File 1: CARE Checklist of information to include when writing this case report.
